# On Turpentine in Puerperal Peritonitis

**Published:** 1817-12

**Authors:** Richard Edgell

**Affiliations:** Surgeon.


*For the London Medical and Physical Journal.*

### *On Turpentine in Puerperal Peritonitis;* by Mr. Richard Edgell, Surgeon.

ABOUT three years ago my attention was strongly excited by the cases published by Dr. Brenan, of Dublin, upon the use of Spirit of Turpentine in the Puerperal Peritonitis; and which were very strongly confirmed by Dr. Douglas. From those cases, it appeared to me that a very reasonable hope might have been entertained of an antidote to this very rapid and frequently-fatal disease. The doctor's medicine had certainly the appearance of a desperate remedy; and, being analogous to no other system, there appeared to be too great a responsibility attached to the practitioner who should be bold enough to adopt it;—and a disregard to the common courtesies of the profession involved him in personal disputes with his neighbours. But, on the other hand, he was borne out by the cases, as far as they went, towards the confirmation of his doctrine. The animosity which existed between him and those professional gentlemen who had the best means of detecting any falsehood, and exposing any fallacy, is now certainly in his favour; inasmuch as no such detection nor exposure has been made.

Let us, therefore, consider how the matter now stands. To say that in proportion as a disease becomes desperate the remedy is interesting, is no more than a truism. That this disease, after its first stages, becomes rapidly desperate, is is evident: how is it, therefore, that this mode of treatment has not excited greater interest? When it was first exhibited, it was considered as adding fuel to fire; and an explosion was expected both of the patient and the system. But so far was it from this, that the fever was allayed, and the inflammation dissipated. This extraordinary fact is so contrary to all theoretical reasoning, *à priori*, that it is of itself sufficient to dispel prejudice and encourage hope. In cases of peritonitis, it very frequently happens, that in the usual mode of treatment, after every expedient has been exhausted, the inflammation terminates rapidly in gangrene. Certainly, under these circumstances, and on the near approach to this crisis, this powerful remedy may be resorted to, on the faith of the reported cases, by the most cautious practitioner. Under these considerations, I have adopted the use of spirit of turpentine. In your Number for May, 1816, I communicated a case of puerperal peritonitis, which appeared to me to be strongly in favour of the use of spirits of turpentine; and I will now beg leave to state a case within my own practice, which, besides its relation to the subject in question, is deserving of notice, from a singular recurrence of the disease some time after it had been dispelled.

In the month of January last, I attended a lady who was attacked on the third day after delivery with peritonitis, which gave way to the following treatment, viz. two large bleedings, and a powder containing fifteen grains of calomel, followed by a draught of infusion of senna with sulphate of magnesia; and the spirit of turpentine was employed at the same time, as a liniment, over the region of the uterus. In the third week after delivery, the patient was again attacked by the same disease. The usual means of depletion by repeated bleedings, generally and locally, each time, *ad deliquium*, and the free use of calomel, were resorted to without success. The patient was rapidly sinking, when it was agreed, in consultation with Mr. Lowe, to have recourse to the stimulating system. A drachm of rectified spirit of turpentine was administered every two or three hours, a large blister was applied over the abdomen, and Madeira wine and opium were freely given. From the time of this change of treatment an amendment took place, and the recovery was permanent. The spirit of turpentine, used internally in this case, certainly added to the stimulus, and as certainly did no harm.

Let us now consider, what facts appear to have been established, and what desiderata remain. And first, it is clear, that spirit of turpentine, used either externally or internally, has not, as might at first have been expected, aggravated the inflammation inflammation in puerperal peritonitis, but that, on the contrary, in every case which has been reported, whether used substantively, or in combination with other stimulants, it has been attended with favourable effects. Secondly, that it has prevailed in cases of great extremity, and in which it is probable, that other stimulants would have been insufficient. Thirdly, that its operation has been almost instantaneous, which, in this rapid disease, is a most happy circumstance.

As to further desiderata, first, it is most important, that it should be ascertained in what state of the disease, whether at its commencement, or at any particular period of its progress, the turpentine may be most advantageously used? Secondly, whether it should follow a general, or what, system of depletion? Thirdly, whether the internal or external application of the medicine be most adviseable, and in what cases? Fourthly, whether the external use of the spirit may not have a reference to Dr. Sutton's suggestion of infrigidation, or of the refrigerative plan of treatment? Lastly, whether the turpentine, or any other successful mode of treatment in the puerperal peritonitis, is not equally, or how far, efficacious in all other cases of inflammation of the peritoneum?

Much has been done under an undefined administration of the medicine; how much, therefore, may be reasonably expected, when its properties shall have been accurately explained!

I have been stimulated to these reflections and observations by the recent occurrence of several cases in this city of peritonitis, which have terminated fatally, although every thing was done which experience could suggest according to the usual mode of treatment, but without the aid of turpentine. As far as my own experience and information have gone, I think that the medicine ought to be resorted to in extreme cases. If it is to be exploded, it should be upon authorities and reasons at least equivalent to those which have been given for its adoption.

*Bristol; Sept.* 22, 1817.

