# Errata

**Published:** 1818-02

**Authors:** 


ERRATA *in the last Volume.*—Page 450, line 10, for *arrangements* read *derangements;* p. 451, line 3, dele *comma* after *corpora;* same page, line 14 from the bottom, and also p. 452, line 35, for *Hydalodes* read *Hydatodes.*

